# The role of the molecular chaperone CCT in protein folding and mediation of cytoskeleton-associated processes: implications for cancer cell biology

**DOI:** 10.1007/s12192-018-0949-3

**Published:** 2018-12-01

**Authors:** Josefine Vallin, Julie Grantham

**Affiliations:** Department of Chemistry and Molecular Biology, University of Gothenburg, 40530 Gothenburg, Sweden

**Keywords:** Actin, Tubulin, Chaperonin, Cancer, Cytoskeleton

## Abstract

The chaperonin-containing tailless complex polypeptide 1 (CCT) is required in vivo for the folding of newly synthesized tubulin and actin proteins and is thus intrinsically connected to all cellular processes that rely on the microtubule and actin filament components of the cytoskeleton, both of which are highly regulated and dynamic assemblies. In addition to CCT acting as a protein folding oligomer, further modes of CCT action mediated either by the CCT oligomer itself or via CCT subunits in their monomeric forms can influence processes associated with assembled actin filaments and microtubules. Thus, there is an extended functional role for CCT with regard to its major folding substrates with a complex interplay between CCT as folding machine for tubulin/actin and as a modulator of processes involving the assembled cytoskeleton. As cell division, directed cell migration, and invasion are major drivers of cancer development and rely on the microtubule and actin filament components of the cytoskeleton, CCT activity is fundamentally linked to cancer. Furthermore, the CCT oligomer also folds proteins connected to cell cycle progression and interacts with several other proteins that are linked to cancer such as tumor-suppressor proteins and regulators of the cytoskeleton, while CCT monomer function can influence cell migration. Thus, understanding CCT activity is important for many aspects of cancer cell biology and may reveal new ways to target tumor growth and invasion.

## Introduction

**Fig. 1:**
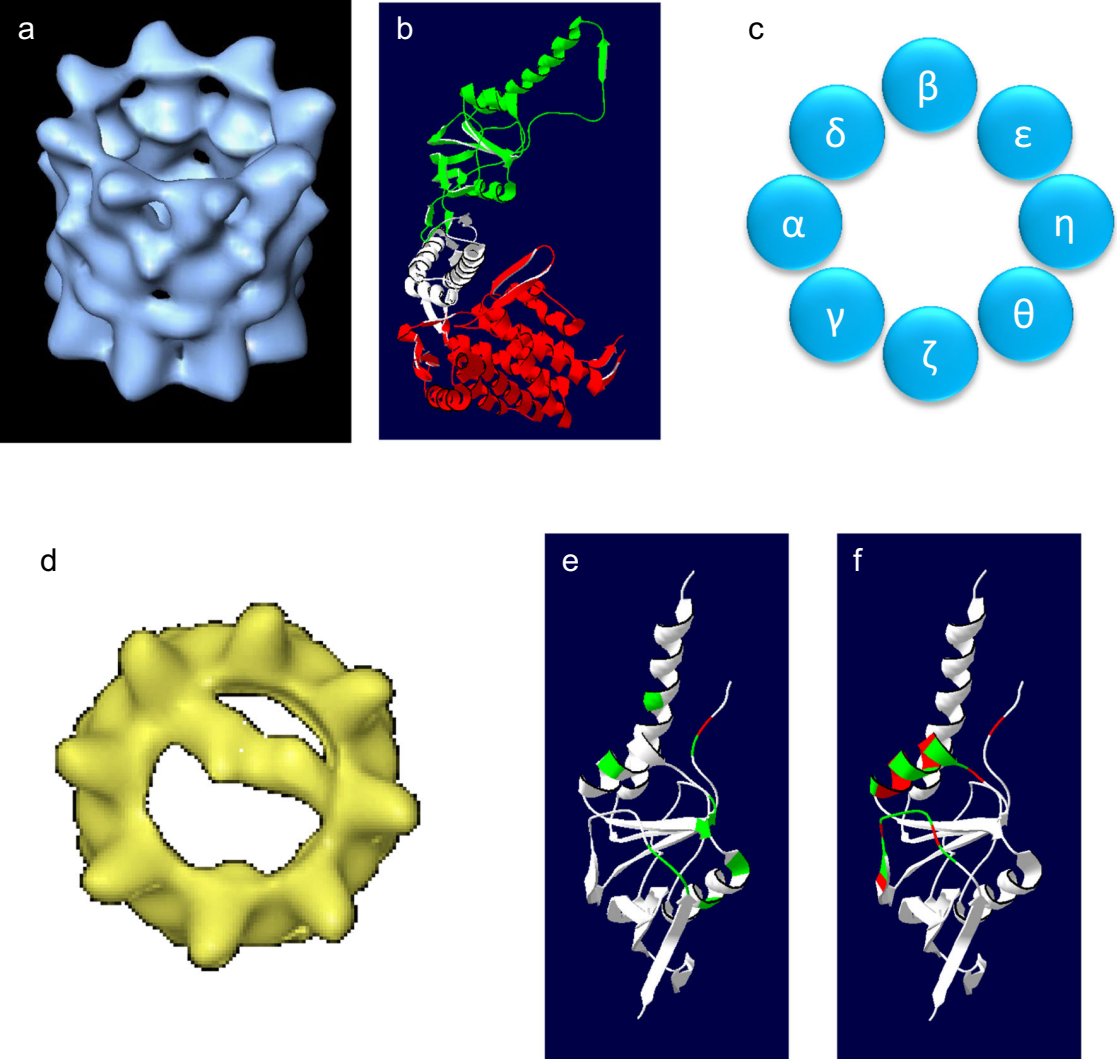
Structure of the CCT oligomer. **a** A three-dimensional reconstruction of the CCT oligomer following cryo-electron microscopy (Llorca et al. 2001). **b** Domain structure of CCT based on the structure of the thermosome (PDB: 1A6D) with the equatorial domain (red), the flexible linker (white), and the apical, substrate-binding domain (green) indicated. **c** The order of the CCT subunits within one chaperonin ring (Kalisman et al. 2012; Leitner et al. 2012). **d** A three-dimensional reconstruction of CCT-actin complexes (Llorca et al. 1999). **e** The signature residues of CCTγ identified by Pappenberger et al. (2002) mapped onto the structure of the apical domain of CCTγ (PDB: 1GN1). Hydrophobic residues are shown in red, others in green. **f** The putative substrate-binding site of CCTγ identified by Joachimiak et al. (2014) mapped onto the structure of the CCTγ apical domain. Hydrophobic residues are shown in red, others in green

Chaperonin-containing tailless complex polypeptide 1 (CCT), also known as tailless complex polypeptide 1 ring complex (TRiC), is a molecular chaperone found in the cytoplasm of all eukaryotes. Since CCT was first identified as a chaperone required for the folding of the major cytoskeletal proteins actin and tubulin (Sternlicht et al. 1993), there has been much debate regarding the range of CCT substrates and mechanisms of action. As a member of the chaperonin family of molecular chaperones, CCT forms a barrel-like structure where two back-to-back rings of subunits surround a central cavity (Fig. 1a). Each ring contains eight subunits, encoded by individual genes, which are essential in yeast (reviewed by Stoldt et al. 1996), named CCTα to CCTθ in mammalian cells and CCT1 to CCT8 in yeast. In addition to CCT, the chaperonin family includes the extensively studied GroEL found in bacteria, Hsp60 found in mitochondria and chloroplasts, and the chaperonins of archaea, such as the thermosome. The chaperonins can be classified as being type I or type II chaperonins based on sequence homology. The former group includes those chaperonins from bacteria and endosymbiotic organelles, which consist of two rings of seven identical subunits, where access to the central cavity can be restricted by the binding of a co-chaperone (GroES or Hsp10). The latter group consists of chaperonins from archaea and the eukaryotic CCT, each of which has a more complex subunit composition (one to three types of subunit for archaea and eight for CCT) and access to the central cavity is controlled by a helical protrusion extending from the apical domain of each subunit.

All chaperonin subunits share the same general domain architecture. The equatorial domain contains an ATP-binding site and is connected via a flexible linker region to the apical, substrate-binding domain (Fig. 1b). In the case of CCT, the equatorial domains of the eight subunits display high sequence homology, while the apical substrate-binding domains have the most divergent sequences between subunits (Kim et al. 1994). Thus, CCT has a subunit composition that is unique amongst chaperonins. The subunit complexity and the need for determining the placement of each subunit within the chaperonin rings have been a major challenge for understanding CCT function. Early conflicting models of the CCT subunit arrangement within the chaperonin rings were based on the composition of CCT micro-complexes of two or more CCT subunits (Liou and Willison 1997), or on cryo-electron microscopy (Cong et al. 2010). The complex nature of the subunit arrangement and size of the CCT oligomer presented a challenge for obtaining high-resolution crystallography data, although the structure of the CCT oligomer bound to actin has been solved at a resolution of 3.8 Å (Dekker et al. 2011) and with tubulin bound at a resolution of 5.5 Å (Munoz et al. 2011). However, a conclusive resolution of the CCT subunit order (consistent for both bovine and yeast CCT) was achieved using the approach of chemical cross-linking followed by mass spectrometry to identify neighboring subunits (Kalisman et al. 2012; Leitner et al. 2012) (Fig. 1c). It is thus necessary to revisit mechanistic studies published prior to these two articles and reassign subunit identities. Here, we will discuss the mechanisms of action of CCT, taking into account both early and more recent studies, as well as giving an overview of CCT activity and the impact of CCT on cellular functions with a focus on cancer cell biology.

## Substrate recognition

Unlike the other chaperonins, CCT consists of eight distinct protein subunits. This creates a unique and complex interaction surface for substrates to bind to, where the geometry of substrate interactions can be determined via the position of CCT subunit-specific binding sites. A major contribution to understanding how CCT interacts with its substrates came from cryo-electron microscopy and single-particle reconstructions of CCT oligomer bound to full-length actin (Llorca et al. 1999) and bound to full-length tubulin (Llorca et al. 2001; Llorca et al. 2000). This approach revealed that actin and tubulin bind directly to several subunits at once, resulting in both folding substrates spanning the central chaperonin cavity. Both actin and tubulin appear to interact with CCT when partially folded and are described by Llorca et al. (2000) as being in “open quasi-native” conformations. Actin interacts with two CCT subunits in a 1.4 orientation (where the numbering is based on the position of the subunits within the ring, Fig. 1d and Llorca et al. 1999) while tubulin has an approximate 1.5 orientation with up to five CCT subunits involved in the interaction (Llorca et al. 2000). Thus, there is a geometry-specific component to the CCT-substrate interaction that can be determined by substrates interacting with specific CCT subunits.

The observations of subunit specificity raise the question of what is the nature of the CCT substrate-binding sites. Pappenberger et al. (2002) identified conserved signature residues for each of the CCT subunits with the reasoning that high levels of conservation will be linked to function. The signature residues for the CCTγ apical domain are illustrated in Fig. 1e and are predominantly on the inner face of the apical domain with only one, Y247, being hydrophobic. Based on the crystal structure of the apical domain of CCTγ, Pappenberger et al. (2002) suggested that interactions between CCTγ and tubulin utilize a combination of polar and electrostatic side chains. Joachimiak et al. (2014) applied a structural approach, combined with biochemical analyses, to assess the binding of human immunodeficiency virus (HIV) protein 6 to the apical domain of CCT3 and a peptide from the Box 1 region of the von Hippel-Lindau (VHL) tumor-suppressor protein to the apical domain of CCT1. They identified a helix and proximal loop on the inside face of the apical domain as being the substrate-binding site with a contribution from Y247, which is located in the hinge of the helical protrusion (Joachimiak et al. 2014) (Fig. 1f). In the case of HIV protein 6 binding to CCT3, Joachimiak et al. (2014) identified an interaction core of consisting of residues L299, H302, M305, Q301, and Y247 in CCT3, two of which (H302 and Y247) are considered to be signature residues according to Pappenberger et al. (2002). Thus, the nature of the substrate interaction surface of a CCT subunit is not dominated by hydrophobic residues and is thus distinct from the bacterial chaperonin GroEL where interactions with substrates would be expected to be predominantly hydrophobic (Chen and Sigler 1999). In the case of GroEL, such binding is consistent with GroEL being able to recognize a wide range of unfolded substrate proteins, while in the case of CCT, substrate specificity would be conferred by specific binding sites. Joachimiak et al. (2014) discuss how binding sites that combine hydrophobic and non-hydrophobic interactions provide “dual recognition” where charge-charge interactions could be involved in the orientation of the substrate and provide specificity. They suggest that binding of substrate to CCT is relatively weak and would thus support multiple binding sites being employed.

The range of proteins that use CCT for their folding has been a topic of much debate: does CCT have a broad range of substrates or is it rather restricted? This question is the focus of a recent review article by Willison (2018) so will not be dealt with in detail here. However, the complexity of the CCT binding interface and the nature of subunit-substrate interactions, together with CCT not being heat stress-inducible, are consistent with CCT being an essential folding component for a rather discrete subset of folding substrates where actin and tubulin isoforms represent the major CCT substrates. As actin and tubulin are abundant proteins and are known to be the major co-precipitating proteins in CCT immunoprecipitation experiments (Grantham et al. 2006), it is important to note that the frequently quoted notion that CCT is folding in the region of 10 to 15% of proteins is likely to be an over-estimation. These numbers are based on the work of Thulasiraman et al. (1999), where newly synthesized proteins were radio-labeled, then immunoprecipitations performed to assess the levels of proteins bound to CCT (by calculating the counts from CCT-bound proteins as a percentage of total counts). As actin and tubulin are very abundant proteins, they alone contribute to a substantial percentage of the radio-labeled proteins bound to CCT (Thulasiraman et al. 1999), and thus, the percentage of counts does not necessarily correlate to the percentage of total proteins.

## Mechanisms of folding, insights from new structural data

**Fig. 2:**
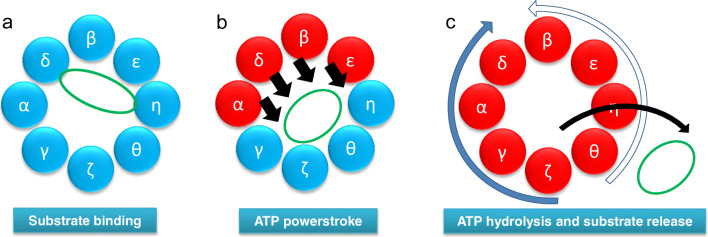
Model of the CCT folding cycle. **a** Actin (green) binds to the CCT oligomer in a 1.4 orientation. **b** ATP binding to high-affinity CCT subunits (red) leads to a powerstroke (black arrows) (Reissmann et al. 2012) and the actin molecule being released from one side of the chaperonin ring (Llorca et al. 2001). **c** After all CCT subunits have bound ATP, a sequential wave of ATP hydrolysis occurs either starting at CCTζ and proceeding clockwise around the ring (solid blue arrow, most probable) or starting at CCTθ and proceeding anti-clockwise around the ring (open blue arrow, less probable) (Gruber et al. 2017). Such a wave of ATP hydrolysis could be coupled to the ordered release of the folding substrate

The solving of the CCT subunit order within the chaperonin rings (Kalisman et al. 2012; Leitner et al. 2012) together with an assessment of the ATP-binding affinities for each of the CCT subunits (Reissmann et al. 2012) has revealed that the four CCT subunits with high ATP-binding affinities (CCTα, β, δ, and ε) are grouped together in the chaperonin ring. This indicates that there is a potential ATP-binding asymmetry within one chaperonin ring. As Llorca et al. (1999) suggest that initial binding of actin to CCT occurs via actin subdomain 4 (L178 to F262 in human β-actin) to CCT subunits that are opposite to CCTδ in a 1.4 orientation, it is probable that initial binding occurs via CCTζ or CCTη while in their APO or ADP conformation (Fig. 2a). This is consistent with CCTζ and CCTη having a low affinity for ATP (Reissmann et al. 2012) and thus may be more likely to be in a non-ATP-bound state at physiological levels of ATP. Indeed, Reissmann et al. (2012) observe that in 1 mM α-[^32^P]-ATP only seven ATP molecules bind to the CCT oligomer (16-mer), which could correspond to most of the high ATP affinity CCT subunits from each ring binding to ATP, rather than all the subunits within a single ring.

ATP binding to CCTα, CCTδ, CCTβ, and CCTε would then generate conformational changes driving a power stroke movement (Reissmann et al. 2012). Such a movement could push the actin molecule towards the opposite side of the chaperonin ring (Fig. 2b), consistent with the structures of actin-CCT and tubulin-CCT complexes in the presence of the non-hydrolyzable analogue of ATP adenylylimidodiphosphate (AMP-PNP), observed by Llorca et al. (2001) where the substrate is observed to have become more compact and no longer spanning across the chaperonin cavity. Using the Arrhenius analysis, Gruber et al. (2017) describe a model where ATP hydrolysis occurs in a sequential manner, possibly as two waves that initiate from CCTζ and CCTθ and proceed around the chaperonin ring in opposite directions (Fig. 2c). This could lead to substrate release where specific points of interaction with substrate can be released sequentially (Gruber et al. 2017) and is consistent with the models presented by Llorca et al. (2001).

With regard to substrate binding and processing, a similar scenario appears to be the case for tubulin. Both the electron microscopy of Llorca et al. (2000) and the cross-linking data presented by Joachimiak et al. (2014) support a model where tubulin spans the cavity of CCT interacting with both a cluster of high ATP affinity CCT subunits and a cluster of low ATP affinity CCT subunits. As previously suggested (Grantham 2010), those substrates that rely upon CCT for folding where specific geometric components of binding are required would be expected to bind to CCT via more than one binding site, thus providing CCT with a mechanical advantage to exert force upon its substrate. In this situation, CCT can be viewed as a “molecular clamp” where the conformational changes within the CCT subunits, driven by the nucleotide cycle, are able to apply mechanical force to the substrate to overcome energy barriers in their folding pathways. Thus, rather than the obligate substrates sharing a common fold that requires interactions with CCT for folding (note that the structures of actin and tubulin are not similar), obligate substrates utilize geometrically specific binding conformations in order to receive mechanical input from the nucleotide-driven folding cycle of CCT.

## CCT oligomer folding activity

Analysis of the CCT oligomer interactome (e.g., Dekker et al. 2008; Yam et al. 2008) will identify folding substrates that are dependent upon CCT for folding (the obligate substrates), proteins that may bind to CCT if off-pathway folding intermediates are formed, proteins that are regulated by CCT, and proteins that regulate CCT function.

### Consequences of misfolding or altered rates of folding

For the obligate substrates, their functions are intrinsically linked to CCT folding activity: if CCT fails to fold such a substrate correctly, then effects from the loss of function of the substrate could occur. Therefore, Cdh1 and Cdc20 both connect CCT function to cell cycle progression (Camasses et al. 2003) and actin and tubulin (Sternlicht et al. 1993) connect CCT to any process that depends on functional microtubules and actin filaments. Additionally, failure to fold substrate proteins could lead to a toxic gain of function where toxicity could arise from the formation of aggregates and misfolded proteins. A study on mutations in cardiac actin that are associated with heart disease found that although the actin mutants could be folded by CCT, there was a substantial impact upon their folding efficiency (Vang et al. 2005). The consequences for cells expressing such mutants are complex. Firstly, the mutant actin proteins could have a direct negative impact on the functional integrity of the actin filaments and secondly, failure to fold efficiently could lead to an accumulation of misfolded proteins. In the case of tubulin, the R264C mutation in α-tubulin, which is associated with pachygyria, is known to be folded more slowly by CCT and also to have limited associations with tubulin co-factor B (Tian et al. 2008). This could potentially lead to a limitation in tubulin dimers and subsequent reductions in microtubule levels that could affect neuronal migration (Tian et al. 2008). With both actin and tubulin, it is possible that mutant proteins that fold more slowly could also disrupt the availability for CCT to interact with other folding substrates, potentially leading to other proteins misfolding and thus have a negative impact upon cellular health.

### Effects of reducing CCT levels

Using siRNA to deplete levels of CCT subunits has the potential to disrupt both CCT oligomer functions and functions associated with specific CCT subunits in their monomeric forms. When one CCT subunit is targeted for depletion, a reduction in the levels of assembled oligomer occurs, which results in an increase in the non-targeted CCT subunits being present as monomers (Brackley and Grantham 2010; Grantham et al. 2006). Therefore, if the targeting of several CCT subunits gives the same results, then it is probable that CCT oligomer function has been affected. However, if the targeting of one CCT subunit gives a unique outcome, then the possibility of a monomer-related function should be addressed.

In cultured mammalian cells, siRNA depletion of either CCTβ, CCTδ, or CCTζ leads to growth arrest, while microinjection with an antibody to CCT, which reduces the rate of actin and tubulin folding by CCT, leads to a delay in S phase progression (Grantham et al. 2006). This is consistent with CCT oligomer function being affected and it is thus not surprising that reducing the levels of the CCTγ subunit is sufficient to reduce cell proliferation (Shi et al. 2018; Zhang et al. 2016). The reduction of CCT levels in *Caenorhabditis elegans* affects microtubule-mediated processes during development (Lundin et al. 2008) and the depletion of CCT5 in *C. elegans* effects the structure of the apical plasma membrane in the microvilli of intestinal cells and results in the formation of actin aggregates (Saegusa et al. 2014). Together, these studies show the importance of CCT activity for the folding of actin and tubulin during the development of a whole animal. Saegusa et al. (2014) also show that when depleting CCT in *C. elegans*, while there is no reduction in total actin levels, tubulin levels are decreased. This is consistent with the observations in cultured mammalian cells where upon CCT depletion, there is little change in total actin levels with actin forming aggregate-like structures, but large reductions in tubulin levels are seen (Grantham et al. 2006).

## CCT activity extends beyond actin and tubulin folding to include associations with actin filaments and microtubules

**Fig. 3:**
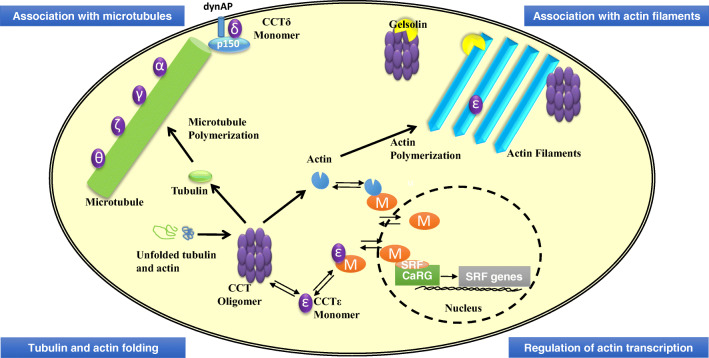
The complex interplay between CCT and actin and tubulin. Cartoon of a eukaryotic cell depicting the interactions between CCT and the actin- and tubulin-based cytoskeletal systems. Tubulin and actin folding. The CCT oligomer folds newly synthesized tubulin and actin (Sternlicht et al. 1993). Regulation of actin transcription. The CCTε subunit when monomeric can act as a component of the SRF pathway by interacting with the co-transcriptional activator MRTF-A (M) and thus has the potential to connect the folding capacity of the cell for actin to the transcription of actin (Elliott et al. 2015). MRTF-A is shown in the nucleus binding together with SRF to DNA sequences containing a CARG motif to initiate the transcription of the SRF genes that include actin and several actin-binding proteins (Sun et al. 2006; Vartiainen et al. 2007). Association with actin filaments. CCTε can associate with actin filament bundles and its levels as a monomer are linked to cell shape (Brackley and Grantham 2010). The CCT oligomer can affect the initial rate of actin polymerization but not the final levels of actin filaments in vitro (Grantham et al. 2002). The actin filament severing and capping protein gelsolin, in its Ca^2+^-bound conformation, binds to the CCT oligomer (Svanstrom and Grantham 2016) but is not a folding substrate of CCT (Brackley and Grantham 2011). Association with microtubules. Some CCT subunits behave as microtubule-associated proteins in vitro (Roobol et al. 1999). CCTδ monomer interacts with p150^Glued^ (a component of the dynactin complex linking the dynein motor to microtubules) in close proximity to the plasma membrane (Echbarthi et al. 2018)

In addition to CCT being required for the folding of actin and tubulin, CCT activity is now known to extend beyond protein folding to include interactions that involve actin filaments and microtubules and also other proteins that are associated with the cytoskeleton. This extended role applies both to the CCT oligomer and to some CCT subunits when in their monomeric forms and is summarized in Fig. 3.

### The CCT oligomer and actin polymerization

The CCT oligomer can interact with actin filaments, reducing the rate of actin polymerization, but not final levels of actin filaments, in in vitro polymerization assays potentially by acting at the plus end of the actin filaments (Grantham et al. 2002). This may reflect an additional need for the chaperoning of actin during the process of polymerization where it is “salt-activated” actin monomers that polymerize. An additional way in which CCT may be able to affect actin polymerization is via the actin filament severing and capping protein gelsolin. Gelsolin, when in its Ca^2+^-activated form, binds to the CCT oligomer (Svanstrom and Grantham 2016) but does not behave as a folding substrate of CCT (Brackley and Grantham 2011). This suggests that CCT has a role in the regulation of gelsolin activity, possibly acting as a sequestering protein for gelsolin, as CCT is able to inhibit actin filament severing by gelsolin in an in vitro assay (Svanstrom and Grantham 2016) and adds a further level of complexity to the interplay between CCT and the cytoskeleton.

### CCT monomer functions with regard to actin/actin filaments

The serum response factor (SRF) pathway connects cell surface signaling with actin transcription (Sotiropoulos et al. 1999). The CCTε subunit (when monomeric) can bind to the myocardin-related transcription factor A (MRTF-A), the co-transcriptional activator of the SRF pathway, and may provide a way to ensure cells only proceed with increasing levels of actin translation in response to cell surface signaling when there is sufficient CCT oligomer to fold the newly synthesized actin (Elliott et al. 2015). The CCTε monomer also associates with actin bundles in cultured mammalian cells and can affect cell shape such that when levels of CCTε are reduced, cells become narrow, while cells with increased levels of CCTε monomer spread out (Brackley and Grantham 2010). This latter observation is an example of a set of siRNA experiments targeting each of the eight CCT subunits individually revealing a monomeric function. In this case, only the depletion of CCTε resulted in cell narrowing, while depleting the other CCT subunits resulted in cell spreading. As the loss of assembled CCT oligomer would occur in all eight subunit depletions, such differences in cell shape cannot be attributed to the loss of the CCT oligomer.

Therefore, in addition to the folding of newly synthesized actin, CCT has the potential to affect both actin transcription (via CCTε-MRTF-A interactions) and assembled actin filaments, either directly or via the modulation of gelsolin activity.

### CCT monomer functions with regard to microtubules

In the case of microtubules, four CCT subunits (CCTα, CCTγ, CCTζ, and CCTθ) act as microtubule-associated proteins in in vitro microtubule assembly assays (Roobol et al. 1999). Consistent with these observations, in cells where CCTε or CCTζ are depleted by siRNA, leading to an increase in the levels of the non-targeted CCT subunits as monomers, there is an increase in the regrowth of microtubules after treatment with the microtubule depolymerization drug nocodazole (Brackley and Grantham 2010). However, in this case, it was not possible to exclude that the loss of the CCT oligomer was a contributing factor.

The fusion of green fluorescent protein (GFP) to CCT subunits in order to hinder their incorporation into the CCT oligomer was used to screen for the effects of CCT monomer over-expression in cultured mammalian cells, where it was found that GFP-CCTδ expression resulted in the formation of retraction fibers (Spiess et al. 2015). The component of the dynactin complex, p150^Glued^, was recently identified as a binding partner for monomeric CCTδ in a yeast two-hybrid screen designed to identify CCTδ monomer binding partners (Echbarthi et al. 2018). The dynactin complex mediates the movement of the dynein motor along microtubules and is thus important for microtubule minus end-directed transport (e.g., Dixit et al. 2008). The binding of p150^Glued^ to CCTδ, together with the transmembrane dynein-associated protein (dynAP), was shown to be important for conferring the previously reported plasma membrane association of GFP-CCTδ and the formation of retraction fibers (Echbarthi et al. 2018; Spiess et al. 2015). Thus, CCTδ may play a role in dynein-mediated transport along microtubules. In the cases of CCT subunits having independent functions when monomeric, it is possible that their role could be to act as sequestering proteins, as may be the case for CCTε and MRTF-A (Elliott et al. 2015). Alternatively, the CCT monomers play a more active functional role. This latter scenario may well be the case for the association of CCTδ with p150^Glued^ as GFP-CCTδ expression results in some p150^Glued^ localizing to the cell periphery. However, a mutation in the ATP-binding pocket of GFP-CCTδ failed to induce the formation of retraction fibers and did not induce p150^Glued^ localization to the plasma membrane, despite binding to p150^Glued^ to the same extent as wild-type GFP-CCTδ (Echbarthi et al. 2018; Spiess et al. 2015). These observations are consistent with CCTδ not only binding to p150^Glued^, but that the ATPase activity of the CCTδ is required for monomer function suggesting that the role of CCTδ may be to actively induce a particular conformation of p150^Glued^.

## Extent of CCT monomer functions

The observations of Amit et al. (2010) where equivalent mutations in the ATP-binding pockets of the eight CCT subunits have differential effects on cellular functions may reflect both hierarchical roles of CCT subunits within the CCT oligomer and also possibly CCT subunit-specific monomer functions. Furthermore, Matalon et al. (2014) show that despite the CCT oligomer containing stoichiometric levels of CCT subunits, this is not reflected in the levels of the individual CCT subunits with increased amounts of CCT4 and CCT8 (equivalent to CCTδ and CCTθ) being found in yeast. Thus, it is probable that more CCT subunits will be found to possess individual functions when in their monomeric states.

How might a monomeric CCT subunit be active? In the case of CCTζ monomer, chaperone-like functions are thought to suppress the phenotypes arising from either over-expression of proteins or expression of mutant proteins (Kabir et al. 2005) and over-expression of CCT1 and CCT4 can effect polyglutamine aggregation (Tam et al. 2006). In both these examples, the CCT subunits could be providing a stabilizing interface for misfolded proteins to bind to. However, with regard to the examples of CCT subunits as monomers being involved in the actin and tubulin systems, these functions appeared to occur via various mechanisms. This could include conferring stability (possibly in the case of some CCT subunits being microtubule-associated proteins and increased CCT monomer levels enhancing microtubule regrowth) (Brackley and Grantham 2010; Roobol et al. 1999), having an active function (in the case of CCTδ and p150^Glued^ (Echbarthi et al. 2018)), or sequestering (as is the case for CCTε binding to MRTF-A (Elliott et al. 2015)). In all of these situations, the ratios of assembled-free CCT subunits will be critical for enabling CCT monomer function and the regulation of CCT assembly may act as a determining switch to allow the cell to balance folding requirements with the modulation assembled cytoskeletal structures and associated functions.

## CCT and cancer

The dependency of the major cytoskeletal proteins tubulin and actin upon the CCT oligomer for their folding intrinsically links CCT to cancer cell biology via cell division (formation of the mitotic spindle and segregation of sister chromatids) and cell migration/invasion (traction generation to drive cell motility and determination of directional migration). In this section, we will also discuss the relevance of additional CCT interactions for affecting cell cycle progression, tumor-suppressor proteins, and cell migration to give an overview of the extensive role of CCT in cancer cell biology.

### CCT and cell cycle progression

In addition to CCT subunit levels increasing in cancer cells (Yokota et al. 2001), CCT subunits have been shown to be upregulated during S phase of the cell cycle and expression levels of CCT are linked to cell growth (Yokota et al. 1999). These authors showed an increase in tubulin synthesis around the G1/S transition, presumably correlated with the need to prepare for assembling the mitotic spindle. Consistently, when cells were arrested in G0/G1, no interaction between CCT and tubulin was observed, whereas in early S phase, a CCT-tubulin interaction was detected (Yokota et al. 1999). In addition to CCT-tubulin interactions being of interest for considering the development of new anti-cancer therapeutics, CCT also interacts with other drivers of cell cycle progression, leading to the potential to block various phases of the cell cycle via interrupting CCT activity. Cdc20 and Cdh1 (both obligate CCT folding substrates) are important for the activation of APC/C (anaphase-promoting complex) at the transition from metaphase to anaphase and during G1 (Camasses et al. 2003). CCT has also been shown to play a role in the mitotic checkpoint system by mediating the release of cdc20 from the mitotic checkpoint complex (MCC) and thereby promoting MCC disassembly, resulting in anaphase initiation (Kaisari et al. 2017). Polo-like kinase1 (Plk1) is important during G2 phase of the cell cycle and is an interaction partner and possible folding substrate of CCT (Liu et al. 2005). Reduction in CCT levels results in lower levels of Plk1, indicating the need of CCT for correctly folded Plk1 (Grantham et al. 2006). It is therefore not surprising that reducing CCT levels or affecting its activity disrupts cell cycle progression (Grantham et al. 2006).

### CCT interactions with tumor suppressors and transcription factors

CCT has been shown to be linked to tumor-suppressor proteins: programmed cell death protein 5 (Pdcd5), the von Hippel-Lindau (VHL) tumor-suppressor protein, and p53. Pdcd5 is a tumor-suppressor protein that is an interaction partner, but not a folding substrate, of CCT and can bind to CCT to specifically inhibit β-tubulin folding, potentially by sterically hindering β-tubulin binding to CCT (Tracy et al. 2014). The subsequent inhibition of β-tubulin folding could then have an effect on division and proliferation of cancer cells. CCT is involved in both the folding of the VHL tumor-suppressor protein and the assembly of the VHL-elongin BC complex where VHL needs to be coupled to elongin BC to be able to fold correctly (Feldman et al. 1999; Melville et al. 2003). Furthermore, CCT is known to fold wild-type p53, a frequently mutated tumor-suppressor protein (Trinidad et al. 2013). CCT also binds to signal transducer and activator of transcription 3 (Stat3), which is an oncogenic transcription factor contributing to tumor formation and malignancies, via the CCTγ subunit in an ATP-dependent manner (Kasembeli et al. 2014). Thus, CCT folds proteins that protect cells from cancer, such as VHL and p53, and also Stat3 that would be expected to promote cancer progression.

### Involvement of CCT in cell migration

In addition to actin requiring interactions with the CCT oligomer for folding (Sternlicht et al. 1993), actin polymerization and assembly are tightly controlled by an array of binding proteins (reviewed by Grantham et al. 2012). Rearrangements of actin filaments are of great importance for both normal cell migration and cancer cell migration/invasion, and indeed, several actin-regulating proteins are implicated in cancer cell migration (reviewed by Olson and Sahai 2009), two of which are now known to bind CCT: the p21-activating kinase PAK4 (Zhao et al. 2017) and gelsolin (Brackley and Grantham 2011). PAK4 is responsible for phosphorylation of the neuronal Wiskott-Aldrich syndrome protein (N-WASP), a protein involved in actin organization (Zhao et al. 2017), while gelsolin is an actin filament severing and capping protein (reviewed by Burtnick et al. 2001) that has the potential to increase actin dynamics by enhancing the number of actin filament ends. As CCT binds to gelsolin and possibly acts as a sequesterer for the Ca^2+^-bound active form (Brackley and Grantham 2011; Svanstrom and Grantham 2016), this interaction may be relevant for the effects of gelsolin on cancer progression. The role of gelsolin in cancer cell biology is not yet clearly understood, as gelsolin has been shown to enhance cell motility when over-expressed (Cunningham et al. 1991) and a reduction of gelsolin in some cancer cell lines leads to a decrease in cancer cell invasiveness (Van den Abbeele et al. 2007). However, gelsolin can be epigenetically down-regulated in some cancers (Mielnicki et al. 1999). Thus, the role of gelsolin may differ depending on the cell type or stage of cancer. It is possible that gelsolin sequestering by CCT can affect the number of accessible actin filament ends ready for polymerization and thus, actin dynamics could be linked to the amount of available CCT oligomer.

In a study to identify proteins that are upregulated in cells able to migrate away from a tumor into Matrigel, CCTγ and CCTδ were found to have increased expression (Wang et al. 2004). In the case of CCTδ, this may correlate to observations that cells expressing increased levels of CCTδ monomer (in the form of GFP-CCTδ) migrated differently to cell transfected with GFP-CCTδ mutants and GFP-CCTβ. Relative to these controls, modest levels of GFP-CCTδ enhanced migration but became inhibitory at high expression levels (Echbarthi et al. 2018). Therefore, CCT monomer functions may also play a role in mediating cell migration.

## Future perspectives

As it is clear that CCT is associated with several processes that are fundamental to the progression of cancer, a greater understanding of the mechanisms of CCT action and elucidating the extent of CCT functions will have the potential to provide targets for the development of new anti-cancer therapeutic agents. For example, microtubules are already the target of anti-cancer therapies and inhibiting CCT-tubulin interactions (and thus tubulin folding) may provide a way to combat tumors that have developed resistance to the currently available taxol-based treatments. In addition to considering the potential for targeting CCT oligomer folding activity, CCT monomer functions will potentially become elevated in polyploid tumor cells, as a consequence of an imbalance of expression levels of the eight CCT subunits due to the CCT genes being situated on different chromosomes. Thus, it is important to consider both the impact of CCT oligomer folding upon substrate activity and the effects of increased CCT monomer function in cancer cell biology. CCT could be a challenging target for cancer therapies with regard to specificity and potential side effects arising due to the number of proteins that interact with CCT. However, it is important to note that reduction in CCT levels by siRNA depletion was shown to have a greater effect on the growth of transformed cells compared to non-transformed cells, indicating that transformed cells may have increased requirements for CCT activity (Guest et al. 2015). Furthermore, targeting CCT binding partners to prevent their interactions with either the CCT oligomer or CCT monomers may be an approach to confer specificity and reduce the risk of side effects.
